# Leveraging the outcome of a frontal bone tumor facial reconstruction case by a multimodal approach

**DOI:** 10.1080/23320885.2024.2365174

**Published:** 2024-07-05

**Authors:** Marek Dobke, Frederic J. Kolb, Douglas M. Arm

**Affiliations:** a Department of Surgery, Division of Plastic Surgery, University of CA San Diego, La Jolla, California, USA; b MicroVascular Tissues, Inc, San Diego, California, USA

**Keywords:** Craniofacial reconstruction, wound healing, oncological reconstruction, craniofacial defect, microvascular tissue

## Abstract

The importance of multimodality in the diagnosis and treatment of medical conditions cannot be overemphasized. Herewith a case of facial malignancy encompassing all stages of management and requiring multimodal approaches for diagnosis, oncological treatment, anatomical reconstruction, and ultimately aesthetics and “identity” is presented.

## Introduction

A multimodal approach to chronic or complex conditions deals with risk assessment, classification and systematization of problems, diagnosis, non-invasive and surgical treatment, lifestyle changes, and management of psychosocial factors. It is striking that a review of the literature pertaining to congenital, traumatic or oncological facial defects indicates that multimodality is frequently reflected by emphasizing imaging of defects; sequencing chemotherapy, immunotherapy or radiotherapy; or application of multiple tissue flaps for reconstruction – which is somewhat incomplete [1]. “Longitudinality” of problems as well as final stages of reconstruction requiring restoration of aesthetics and dignity are rarely addressed, or are the subject of separate studies [2–4]. The presented case of facial malignancy encompassing all stages of management and requiring multimodal approaches for diagnosis, oncological treatment, anatomical reconstruction, and ultimately aesthetics and “identity” underscores the importance of maintaining a multimodal perspective throughout treatment.

## Patient

**Figure 1.**
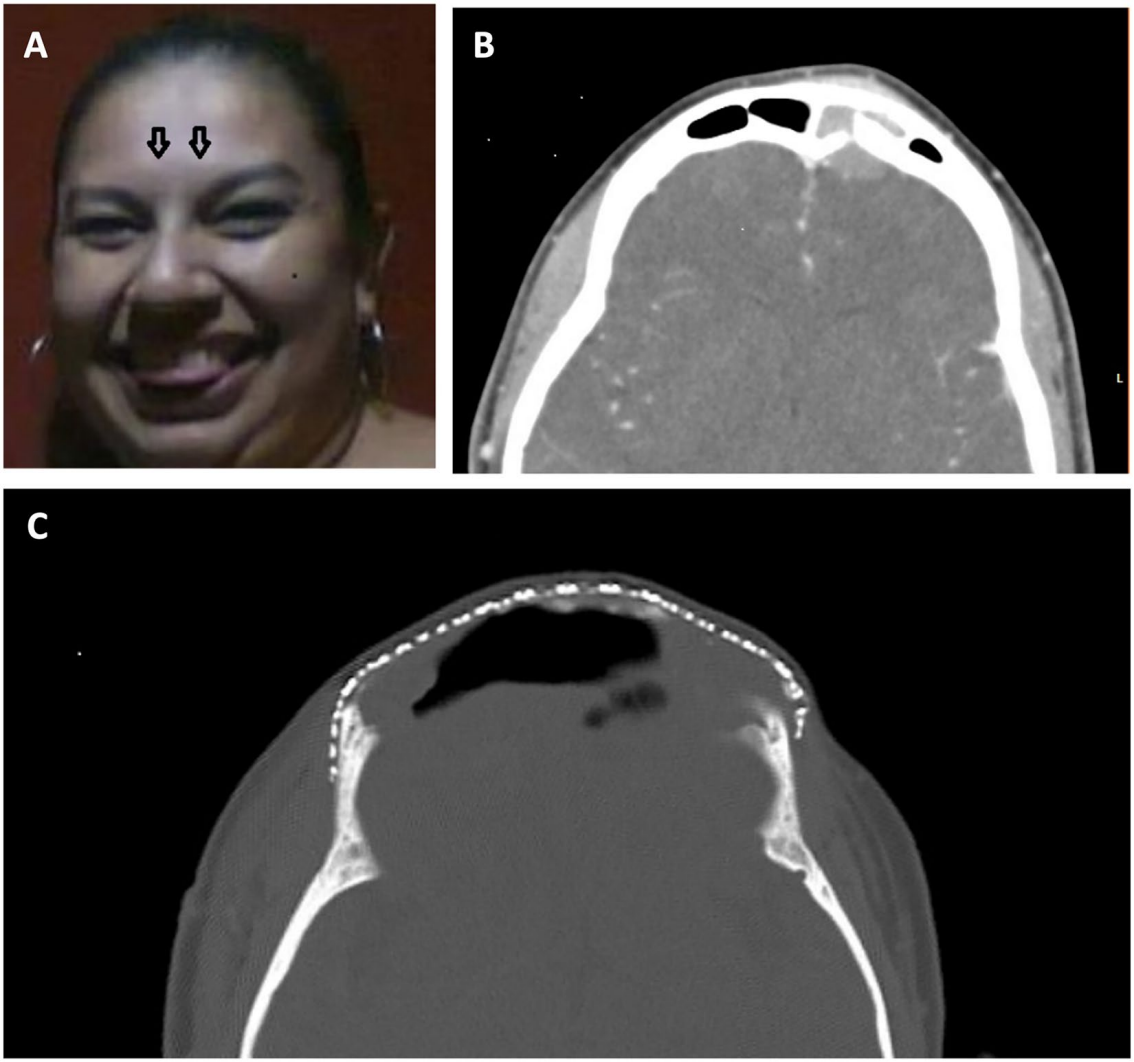
(A) Painful forehead lump (noted in the spring of 2013). (B) Computed tomography demonstrates a left frontal area mass extending through the sinus to the frontal lobe. (C) Status post multiple craniotomies and placement of the titanium mesh (2017).

During her daughter’s quinceañera celebration, a 40-year-old female fell on her face and experienced unusual and non-subsiding mid-forehead pain at the site of parallel “swellings” she hadn’t noticed before (Figure 1A), prompting her to seek medical help.

Computed tomography scans revealed a 2.4 cm diameter enhancing soft tissue mass in the left frontal sinus area extending through eroded inner and outer tables of the calvarium/sinus walls (Figure 1B). Initial diagnoses of possible aggressive infection such as Pott’s puffy tumor or fungal infection with osteomyelitis were considered, although fluid and an enhancing rim indicated an abscess was absent. Neoplasm was not excluded. Extensive sinus disease likely infectious with polyposis was one of the initial diagnoses.

## Materials and methods

Surgery revealed basaloid squamous cell carcinoma (BSCC) of the frontal sinus. Surgery and subsequent chemoradiation were completed in October 2013. In the decade following tumor resection the patient has had a complicated course requiring additional intracranial operations, revisions of her frontal sinus cranialization, drainages of abscesses, and titanium cranioplasties. Complications included pneumococcal meningitis, hydrocephalus, rhinorrhea, and seizures. A ventriculo-peritoneal shunt was placed twice. A few months later she developed recurrent frontal sinusitis, requiring removal of the original frontal fat graft and debridement of the frontal bone flap for epidural infection, with removal of osteomyelitic parts of the frontal bone, and a mixture of CSF and pus (epidural hematoma). In April 2014 she underwent a cranioplasty procedure, during which a large section of titanium mesh, shaped to conform to the frontal convexity and the cranial defect, was implanted (Figure 1C).

**Figure 2.**
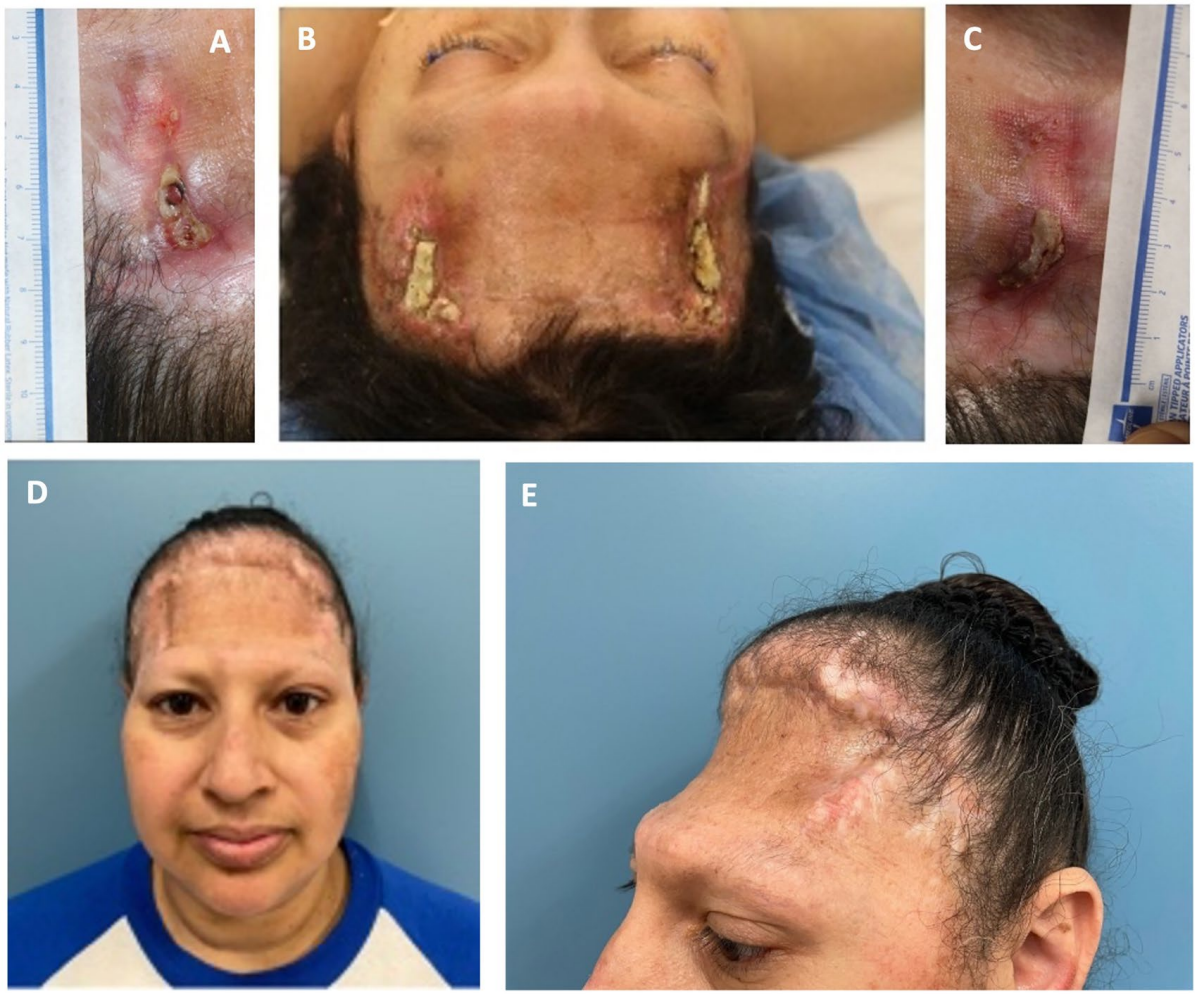
(A–C) Baseline images prior to initial mVASC treatment. At this point, the perimeters of the wounds were lightly debrided to remove necrotic tissue and encourage punctate bleeding. One vial of mVASC was rehydrated in 1.5 cc of sterile water, with equal amounts then injected into the soft tissue immediately surrounding both wound sites. A non-adherent dry dressing (adaptic touch, systagenix, Gatwick, United Kingdom) was applied over the wounds. (D–E) Residual wounds healed after mVASC treatment, following spontaneous separation of necrotic bone sequestra and wound surface reepithelialization.

The patient subsequently developed bilateral soft tissue defects with exposure of necrotic frontal bone lateral “stumps” (Figures 2A–2C). Local skin and temporalis muscle flaps failed to provide adequate, stable coverage.

In this reconstructive stalemate with chronic infection of the frontal bone stumps, post-radiation osteoradionecrosis, and recurrent cerebrospinal fluid leak drainages – all of which posed relative contraindications for free flap-based soft tissue replacement of the entire forehead tissue – angiogenic agents were needed to stabilize the deteriorating defects. mVASC, a human microvascular tissue allograft (MicroVascular Tissues, Inc., San Diego, CA) known to repair deficient microvasculature and improve microvascular perfusion was selected to help heal the residual wounds to minimize surgical risks as well as the ever-present risk of contamination of the titanium mesh during microvascular surgery [5,6]. After three monthly mVASC injections to the defects’ periphery, the wounds healed (Figure 2D–E).

## Results

This adjuvant angiogenic treatment using mVASC microvascular graft for management of the recalcitrant wounds effectively complemented the multimodal treatement for cancer.

**Figure 3.**
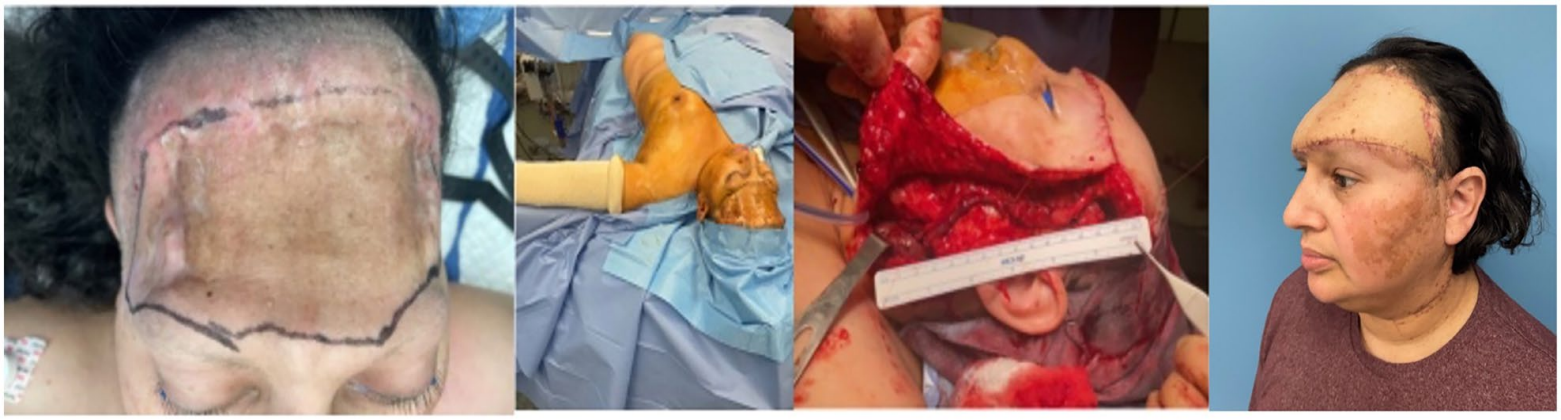
Muscle-sparing lateral chest wall perforator flap, including skin and subcutaneous tissue based on the lateral thoracic artery, was dissected and transferred to the forehead with restoration of flap vascularity by microanastomoses with superficial temporal artery and vein [7]. there was no exposure of the mesh during tangential removal of the scarred skin and layer of subcutaneous tissue.

Following wound closure, when the potential flap recipient area was stable and without recurrent infections, the final stage of reconstructive surgery took place (Figure 3). Irradiated and attenuated native skin was hydrodissected from the underlying dura and removed. Several pieces of frontal bone located bilaterally around the lateral edges of the defects were mechanically debrided. A small, linear 6 mm dural defect was repaired using fine Prolene (Ethicon, Raritan, NJ) suture prior to placement of the soft tissue free flap.

Two-year follow up demonstrated no tissue breakdown, infection or signs of meningitis, or cerebrospinal fluid leak.

**Figure 4.**
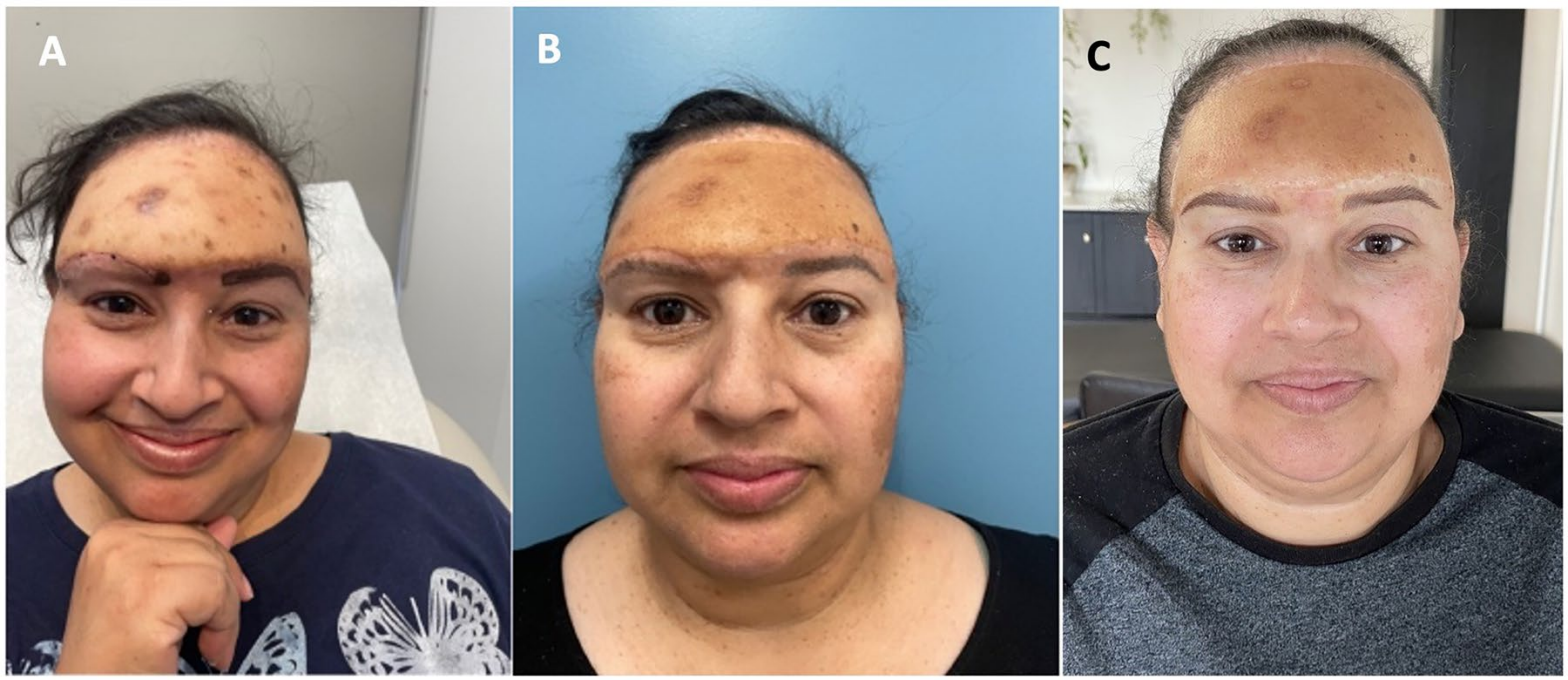
(A) Planning for tattoo-based restoration of the right eyebrow; (B) After two microblading sessions; (C) After hyperpigmentation has subsided (June 2023).

Psychologically essential, self-esteem and dignity-rebuilding cosmetic interventions are vital to complete the treatment. In the presented case, the patient smiled for the first time not after successful free flap reconstruction, but after her right eyebrow recreation and when she was offered lowering of her frontal hairline (Figure 4).

## Discussion and conclusions

The management of frontal sinus malignancies remains a complex entity with no widely accepted algorithm for the timing and invasiveness of interventions. However, a retrospective assessment of final outcomes, interim outcomes and complications indicates that multimodality and “worst-case scenario” plans must be in place at the commencement of treatment. There are few cases of BSCC of the nose and paranasal sinuses reported in literature. BSCC is an aggressive tumor with high rates of nodal (64%) and distant (44%) metastasis. A case-controlled study has shown BSCC has a 6-times higher risk of distant metastasis compared to the usual type of squamous cell carcinoma. Treatment of choice is complete surgical excision and radiotherapy/adjuvant chemotherapy [8].

This case exemplifies that to maximize the success of multimodal cancer therapy involving surgery, radiation, chemo- and immunotherapy, a multimodal approach should also include reconstruction and rehabilitation phases [9–11]. Predisposition for local evolution and recurrence with a severe prognosis of sinusoidal basal squamous cell carcinoma justifies surgical approaches securing the quality of margins as the first objective of treatment with possible “worst-case scenarios” in mind [8,10,11]. Creation of a secure barrier between the nasal cavity and frontonasal duct is one of the key operative tasks in cranialization of the frontal sinus. Variations in the development of fronto-nasal communication with frequent, not easily visible, small connections between the sinus and nasal cavity must be considered. Therefore, this secure anatomical barrier covering and separating frontal lobes, thus allowing placement of mesh implants and reoperation if needed in oncological cases, is one of the key operative tasks [12,13]. The same concept pertains to reconstruction. Retrospective assessment indicates that free fat grafting to separate the sinus cavity from the brain and local soft tissue flaps involving scarred and irradiated tissue for external defect repair in inflamed and poorly vascularized areas were doomed for failure. In anticipation of these morbid problems, it is reasonable to propose technical solutions even though they may be considered overkill before such complications occur. The overall course of the presented case supports the notion that the adequacy of margins and the quality of dural repairs separating the nasal cavity from the brain is essential. In general, endoscopic transnasal surgery or an unnecessarily conservative open approach are less predictable and appear to be oncologically less successful [8–11,13].

In cases such as this where a myriad of challenges to a successful clinical outcome exists, the timely use of an advanced tissue product such as mVASC to restore local blood flow and support rapid closure of the wounds is warranted to minimize infection risk and support the viability of transplanted tissues. Furthermore, additional intracranial operations, frontal sinus cranialization revisions, drainages of abscesses, titanium cranioplasties, pneumococcal meningitis, hydrocephalus, recurrent frontal sinusitis requiring debridement and removal of fat graft and osteomyelitic frontal bone flap for recurrent epidural infection, and such complications as recurrent rhinorrhea and seizures may have been avoided if an aggressive multimodal, multispecialty approach was in place to begin with [8,9].
